# Predictors of Dropouts From a San Diego Diabetes Program: A Case Control Study

**Published:** 2004-09-15

**Authors:** Stephen R. Benoit, Ming Ji, Regina Fleming, Athena Philis-Tsimikas

**Affiliations:** UCSD-SDSU Preventive Medicine Residency, University of California, San Diego Department of Family and Preventive Medicine, La Jolla, Calif. Dr. Benoit is now with the Centers for Disease Control and Prevention; San Diego State Graduate School of Public Health, San Diego, Calif; UCSD-SDSU Preventive Medicine Residency; The Whittier Institute for Diabetes, La Jolla, Calif

## Abstract

**Introduction:**

The objective of this study was to determine the demographic, treatment, clinical, and behavioral factors associated with dropping out of a nurse-based, low-income, multiethnic San Diego diabetes program.

**Methods:**

Data were collected during a 17-month period in 2000 and 2002 on patients with type 2 diabetes from Project Dulce, a disease management program in San Diego County designed to care for an underserved diabetic population. The study sample included 69 cases and 504 controls representing a racial/ethnic mix of 53% Hispanic, 7% black, 16% Asian, 22% white, and 2% other. Logistic regression was used to determine factors associated with patient dropout.

**Results:**

Patients who had high initial clinical indicators including blood pressure and hemoglobin A1c and those who smoked currently or smoked in the past were more likely to drop out of the diabetes program.

**Conclusion:**

This study provides markers of patient dropout in a low-income, multiethnic, type 2 diabetic population. Reasons for dropout in this program can be investigated to prevent further cohort loss.

## Introduction

More than 15 million people have diabetes in the United States, and it is now the sixth leading cause of death (1,2). The United Kingdom Prospective Diabetes Study and Kumamoto Study confirm that improved glucose control reduces the microvascular complications of type 2 diabetes such as retinopathy, nephropathy, and neuropathy (3,4). Because of these findings, new standards of care and new models of health care delivery have emerged to reduce the complications of diabetes and thus improve quality of life (5).

Nonadherence to diabetes treatment strategies prevents patients from meeting optimal standards of care. Jacobsen et al found that up to 34% of patients with type 1 diabetes defaulted from care, and their glycemic control was significantly worse than those who attended their visits (6). Evidence suggests that patients with diabetes who are unable or unwilling to adhere to treatment regimens suffer greater morbidity than those under regular medical supervision (7).

Studies have investigated patients' reasons for non-attendance in diabetes care clinics. Reasons include financial or transportation issues, inability to get time off work, forgetting about appointments, feeling too ill, crowded clinic settings, administrative errors, and feeling the appointment was unnecessary. Other studies have addressed nonadherence in diabetes clinic settings and found significant factors to be smoking, poor education, living long distances from the clinic, and dietary treatments (8).

The purpose of this study was to investigate the factors associated with patients dropping out of Project Dulce, a nurse-based diabetes disease-management system in San Diego, Calif (9). The factors studied included demographic, treatment, clinical, and behavioral variables. The program, started in 1998, is an initiative of Community Health Improvement Partners, the Council of Community Clinics, and The Whittier Institute for Diabetes. Project Dulce uses a multifaceted approach and focuses on providing care to racial/ethnic groups that often lack access to medical services.

## Methods

### Data study sample

Patients with diabetes are referred to Project Dulce by primary care providers. After the patient is referred, a nurse educator conducts an initial assessment and follows the American Diabetes Association (ADA) standards of appropriate physical and laboratory exams and referrals to specialists (e.g., ophthalmologists, podiatrists). The nurse educator is the case manager and follows up on missed patient appointments in addition to identifying individual service and access needs of his or her panel of patients. The nurse also communicates with the primary care physician regarding clinical-care issues. Dieticians are also on staff at Project Dulce to meet with patients referred by the nurse educators. The program is currently active in 17 sites, including community clinics and hospital ambulatory care centers throughout San Diego County. Project Dulce uses the same procedures at each site and tracks patients with diabetes electronic management system (DEMS) software. The database contains demographic, treatment, clinical, and behavioral factors for each patient and collects the information over time. This study included data from July 18, 2000 to October 7, 2002 and was approved by the Institutional Review Board of San Diego State University.

### Eligibility criteria

Patients with type 2 diabetes were first selected from the database. This reduced the population size to 1357 from 1728. Case-control methodology was used in the analysis. We defined cases as patients who dropped out of the program and selected them using the following inclusion criteria: the patient could not have more than two Project Dulce visits; could not be in the program more than three months; needed a baseline A1c value; and the last Project Dulce visit had to be at least six months before October 7, 2002 (the last Project Dulce visit date in the database). The control group consisted of active patients in Project Dulce, and these patients were selected using the following criteria: each patient had to have at least two A1c values at least six months apart, had to be in the program for at least six months, and had to have at least three Project Dulce visits. Eighteen cases (dropouts) and 69 controls were excluded due to missing analytic variables, leaving 69 dropouts and 504 controls.

### Measures and diagnostics

All variables came from the DEMS database and were grouped into five clusters: demographic factors, disease duration, treatment factors, clinical characteristics, and behavioral factors.

The five variables in the demographic cluster were sex, age, race/ethnicity, primary language, and insurance. For purposes of this study, we created five racial/ethnic categories: Hispanic, black, Asian (including Eastern Indian), white, and other.

Most of the patients in Project Dulce have County Medical Services, an insurance program funded by San Diego County to care for the medically indigent adult (MIA) population. However, some patients who surpass maximum income limits or who are not documented are uninsured and pay out-of-pocket to enroll in the program. A smaller proportion of the patients have Medicare, Medicaid, or private insurance. For purposes of this study, insurance status was categorized as uninsured, County Medical Services (representing the MIA population), or insured (including Medicare, Medicaid, or private insurance).

Disease duration was estimated by subtracting the diabetes diagnosis date from the date of the patient's initial Project Dulce visit. The treatment factor cluster was represented by the type of medicine the patient was using at the initial visit. The medicines used for glucose control (e.g., insulin, sulfonylureas, metformin, glitazones, alpha glucosidase inhibitors, meglitinides) were categorized into three levels: insulin alone or insulin with oral agents, more than one oral agent but no insulin, and one oral medication or no medication at all.

Clinical characteristics included baseline systolic (SBP) and diastolic (DBP) blood pressure, Body Mass Index, and baseline A1c. Blood pressure was categorized according to American Diabetes Association standards for optimal control: <130 mm Hg for SBP and <80 mm Hg for DBP (5). The behavioral factor in the model was smoking status. Variables left in the continuous form were assessed for nonlinearity. Collinearity among independent variables was assessed using tolerance values. All tolerance values were substantially greater than 0.10, indicating that collinearity was not an issue.

### Statistical analysis

Variable screening was done using univariate logistic regression with an alpha significance level of 0.25. Those variables significant in univariate analysis were placed in a multivariate model. Variables not significant at the alpha level of 0.05 were assessed as confounders. Each potential confounder was added to the exposures of interest one by one. Parameter estimate changes of greater than 20% were considered significant. Sex, primary language, and baseline DBP were considered confounders and were therefore included in the final model.

## Results

Table 1 shows the descriptive and univariate statistics. There appear to have been fewer Asians and more whites in the dropout group compared with the control group, while the percentage of Hispanics remained consistent among both groups. More of the dropout population (58.6%) spoke English than the control population (47.2%), and more of the dropout population was insured by County Medical Service (60.9%) than the control population (43.5%). Among the dropouts, high baseline blood pressures (defined here as ⩾ 130 mm Hg for SBP and ⩾ 80 mm Hg for DBP) were more common compared to the control population: 46.1% of the dropout population had high SBP compared to 29.5% of the control, and 26.9% of the dropout population had high DBP compared to 11.7% of control. More of the dropouts compared to controls were current smokers (20.3%, dropouts compared to 12.5%, controls) or past smokers (47.3%, dropouts compared to 32.0%, controls).

Table 2 presents the results of the final logistic model. Insurance status, initial blood pressure, baseline A1c, and smoking habit were significant predictors of dropout status. The odds of dropping out of Project Dulce were 95% lower for a patient without insurance compared to a patient with insurance. The odds of being a dropout were 1.8 times higher for a patient with a SBP ⩾130 mm Hg compared to a patient with a SBP <130 mm Hg and 2.3 times higher for a patient with a DBP ⩾80 mm Hg compared to a patient with DBP <80 mm Hg. For every two-unit increase in baseline A1c, the odds of being a dropout increased 1.3 times. Compared with a patient who never smoked, the odds of dropping out of the program were 3.7 times higher for a current smoker and 2.9 times higher for a past smoker. The variables mentioned above were significant after controlling for all other variables in the model (sex, primary language, insurance status, blood pressure, baseline A1c, and smoking status).

## Discussion

Univariate associations between dropouts and controls showed that race/ethnicity, primary language, insurance status, baseline blood pressure, and smoking varied among the two groups. In multivariate analysis, insurance status, baseline blood pressure, baseline A1c, and smoking habit were different between dropouts and controls.

Similar to the findings of Graber et al (10) and Jacobson et al (6), sex and age were not significant predictors of dropout. Other studies have found that men and younger patients are less likely to adhere to treatment (11-13). Race/ethnicity was a predictor of dropout in univariate analysis but lost its significance after controlling for other factors. Dove and Schneider (14) and Goldman et al (11) also found this to be the case. Primary language also lost its significance in multivariate analysis. Disease duration and type of medicine used to treat diabetes did not predict patient adherence to the program. This finding is consistent with Jacobson et al (6) and Graber et al (10) but contradicts Irvine and Mitchell (15).

Insurance status was a significant predictor of patient adherence. The uninsured were 95% less likely to drop out of the program compared to those with insurance. The uninsured pay an enrollment fee to cover nurse visits, laboratory measurements, and diabetes-related medications. They are not eligible for county or federal health services because of documentation status or income limits. The initial monetary investment and lack of affordable health care options are clearly reasons that the uninsured were less likely to drop out. Those with County Medical Services and Medicaid/Medicare do not pay to enroll in Project Dulce and have other provider options for their diabetes care.

Those with high initial blood pressure or A1c were more likely to drop out of the program. These clinical markers may indicate that these patients were more ill and conceivably unable to keep their outpatient appointments. Perhaps their time was occupied with other primary care or specialty visits to address other medical problems. Regardless of the reasons for patient nonadherence, these clinical markers are important. Higher A1c levels put the cases at increased risk for developing microvascular disease, including retinopathy, nephropathy, and neuropathy (3,4). Elevated initial blood pressure put the cases at increased risk of heart attacks, heart failure, strokes, and kidney disease (16).

Smokers that have type 2 diabetes are at even greater risk of micro- and macrovascular disease (17). This study showed that those who smoked or smoked in the past were more likely to drop out of the program. Why these patients are leaving is not clear. Perhaps smokers are less interested in their health, which would explain their lack of interest in Project Dulce. Another possible explanation is that smokers feel rejected by providers that admonish their behavior (10). Smoking could also be a surrogate for life stressors that prevent appointment attendance due to unstable living or financial environments (18). Regardless of the reason, the clinical and behavioral markers that predict nonadherence leave these patients at high risk of morbidity and mortality associated with microvascular and macrovascular disease (19).

This study has limitations. According to Griffin (8), adherence may be less related to demographic factors and more to patients' perceptions, beliefs, and attitudes. Perhaps some patients may not have understood the importance of consistent glucose control, had a different perception of the disease, or were fearful of the multiple medications needed to treat diabetes. Others may have experienced adverse side effects to medication, stopped treatment, and were ashamed to return to their provider. Patient perceptions could explain nonadherence, but none of this information was available for this study.

Similarly, other possible confounders, such as distance living from the clinic, transportation issues, work schedules, whether they knew they had an appointment, stability of living environment, alcohol and drug problems, other comorbid medical/psychological problems, and patient satisfaction and ability to talk with providers, were not available. These factors could also significantly predict dropouts in Project Dulce. Some of the demographic, clinical, and behavioral factors significant in this model may lose statistical significance depending on the importance of the unmeasured predictors. Lastly, 18 dropouts and 69 controls were excluded because of missing data, which could potentially introduce a selection bias. Because patient identifiers were scrambled for privacy prior to copying the dataset, medical records could not be reviewed to recover this data.

In summary, with the available data, this predictor analysis found that Project Dulce patients with higher initial blood pressure readings and A1c values and those who smoked or smoked in the past were more likely to drop out of the program. These results have clinical applicability in that those patients in most need of care were more likely to drop out of the program. Investigating reasons for dropping out would best be done with patient interviews. In this way, patient perceptions, attitudes, and beliefs could be explored in addition to other logistical reasons.

Project Dulce can use the results of this study to minimize future patient loss to follow-up. Although all patients are case-managed by nurses, an intensified approach would be beneficial in those patients with higher initial A1c and blood pressure measurements and in those who smoke or smoked in the past. Acquiring additional information from patients who drop out could help explain the obstacles facing this low-income, multiethnic population. Addressing these obstacles with interventions and/or organizational changes may lead to better clinical outcomes.

## Figures and Tables

**Table 1:** Descriptive and Univariate Statistics to Assess Factors Associated with Dropping Out of a Diabetes Program, San Diego County, 2000–2002^a^,^b^

	**Controls** **N = 573**	**Dropouts** **N = 87**	***P* c**

**Demographic factors**			

**Sex**			.22
** Female**	392 (68.6)	33 (37.9)	
** Male**	179 (31.4)	54 (62.1)	
**Age (mean years)**	573 (55.4)	87 (54.9)	.66
**Race/ethnicity**			**.008**
** Hispanic**	304 (53.3)	45 (51.7)	
** Black**	39 (6.8)	7 (8.1)	
** Asian (includes Eastern Indian)**	100 (17.6)	4 (4.6)	
** White**	116 (20.4)	31 (35.6)	
** Other**	11 (1.9)	0 (0)	
**Primary language**			**.048**
** Not English**	302 (52.8)	36 (41.4)	
** English**	270 (47.2)	51 (58.6)	
**Insurance status**			**<.001 **
** Uninsured**	169 (29.5)	3 (3.5)	
** County Medical Services** ** (Medically Indigent Adults)**	249 (43.5)	53 (60.9)	
** Insurance**	155 (27.0)	31 (35.6)	

**Treatment factor**			

** Medicine**			.28
** Insulin alone or insulin plus oral agents**	102 (17.8)	21 (24.1)	
** >1 oral agent (no insulin)**	210 (36.7)	26 (29.9)	
** No medicine or 1 oral agent**	261 (45.5)	40 (46.0)	

**Clinical characteristics**			

**Baseline systolic blood pressure**			**.003**
** ≥130 mm Hg**	169 (29.5)	36 (46.1)	
** <130 mm Hg**	404 (70.5)	42 (53.9)	
**Baseline diastolic blood pressure**			**<.001**
** ≥80 mm Hg**	67 (11.7)	21 (26.9)	
** <80 mm Hg**	506 (88.3)	57 (73.1)	
**Baseline body mass index (kg/m²) **	557 (32.1)	82 (33.3)	.29
**Baseline A1c**	573 (8.46)	87 (8.80)	.22

**Behavioral factor**			

**Smoking habit**			**.001**
** Current**	63 (12.5)	15 (20.3)	
** Past**	162 (32.0)	35 (47.3)	
** Never**	281 (55.5)	24 (32.4)	
**Disease duration before starting** **Project Dulce (mean years)**	558 (7.08)	83 (7.54)	.59

a Data are given as N (%) except where noted.

b Missing data resulted in case/control sample size differences among analytic variables.

c Statistically significant results are in **boldface**

**Table 2:** Multivariable Logistic Regression Model to Assess Characteristics Associated with Dropping Out of a Diabetes Program, San Diego County, 2000–2002

**Cases (N = 69)** **Controls (N = 504)**	**Odds Ratio**	**(95% CI)^a^**	***P* b**
**Sex**			
** Female**	1.50	(0.83-2.70)	.18
** Male**	1.00	ref^c^	ref
**Primary Language**			
** Not English**	.93	(0.53-1.65)	.81
** English**	1.00	ref	ref
**Insurance status**			**<.001**
** Uninsured**	.05	(0.01-0.24)	**<.001**
** County Medical Services ** ** (Medically Indigent Adults)**	.85	(0.48-1.51)	.58
** Insurance**	1.00	ref	ref
**Baseline systolic blood pressure**			
** ≥130 mm Hg**	1.84	(1.03-3.28)	**.04**
** <130 mm Hg**	1.00	ref	ref
**Baseline diastolic blood pressure**			
** ≥80 mm Hg**	2.26	(1.11-4.58)	**.02**
** <80 mm Hg**	1.00	ref	ref
**Baseline A1c (2% interval)**	1.27	(1.02-1.57)	**.03**
**Smoking habit**			**<.001**
** Current**	3.69	(1.67-8.14)	**.001**
** Past**	2.86	(1.53-5.32)	**.001**
** Never**	1.00	ref	ref

a CI indicates confidence interval.

b Statistically significant results are in **boldface**.

c Ref indicates reference group.
